# Cyclodepsipeptide Biosynthesis in *Hypocreales* Fungi and Sequence Divergence of The **Non-Ribosomal Peptide Synthase Genes**

**DOI:** 10.3390/pathogens9070552

**Published:** 2020-07-09

**Authors:** Monika Urbaniak, Agnieszka Waśkiewicz, Artur Trzebny, Grzegorz Koczyk, Łukasz Stępień

**Affiliations:** 1 Plant-Pathogen Interaction Team, Department of Pathogen Genetics and Plant Resistance, Institute of Plant Genetics of the Polish Academy of Sciences, Strzeszyńska 34, 60-479 Poznań, Poland; lste@igr.poznan.pl; 2 Department of Chemistry, Poznań University of Life Sciences, Wojska Polskiego 75, 60-625 Poznań, Poland; agat@up.poznan.pl; 3 Molecular Biology Techniques Laboratory, Faculty of Biology, Adam Mickiewicz University Poznan, Uniwersytetu Poznańskiego 6, 61–614 Poznan, Poland; arturtrzebny@amu.edu.pl; 4 Functional Evolution of Biological Systems Team, Department of Biometrics and Bioinformatics, Institute of Plant Genetics of the Polish Academy of Sciences, Strzeszyńska 34, 60-479 Poznań, Poland; gkoc@igr.poznan.pl

**Keywords:** *Hypocreales*, cyclodepsipeptides, beauvericin, enniatin, fungi, non-ribosomal peptide synthase

## Abstract

Fungi from the *Hypocreales* order synthesize a range of toxic non-ribosomal cyclic peptides with antimicrobial, insecticidal and cytotoxic activities. Entomopathogenic *Beauveria*, *Isaria* and *Cordyceps* as well as phytopathogenic *Fusarium* spp. are known producers of beauvericins (BEAs), beauvenniatins (BEAEs) or enniatins (ENNs). The compounds are synthesized by beauvericin/enniatin synthase (BEAS/ESYN1), which shows significant sequence divergence among *Hypocreales* members. We investigated ENN, BEA and BEAE production among entomopathogenic (*Beauveria*, *Cordyceps*, *Isaria*) and phytopathogenic (*Fusarium*) fungi; BEA and ENNs were quantified using an LC-MS/MS method. Phylogenetic analysis of partial sequences of putative *BEAS*/*ESYN1* amplicons was also made. Nineteen fungal strains were identified based on sequence analysis of amplified ITS and *tef*-1α regions. BEA was produced by all investigated fungi, with *F. proliferatum* and *F. concentricum* being the most efficient producers. ENNs were synthesized mostly by *F. acuminatum*, *F. avenaceum* and *C. confragosa*. The phylogeny reconstruction suggests that ancestral BEA biosynthesis independently diverged into biosynthesis of other compounds. The divergent positioning of three *Fusarium* isolates raises the possibility of parallel acquisition of cyclic depsipeptide synthases in ancient complexes within *Fusarium* genus. Different fungi have independently evolved NRPS genes involved in depsipeptide biosynthesis, with functional adaptation towards biosynthesis of overlapping yet diversified metabolite profiles.

## 1. Introduction

**Figure 1:**
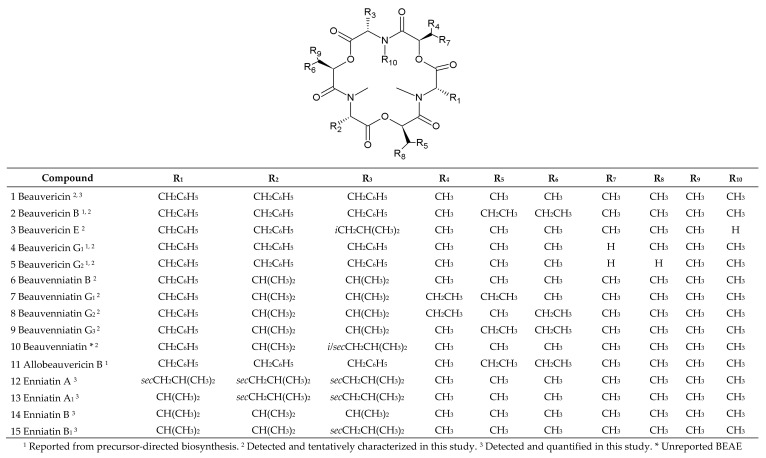
Overview of chemical structures of the beauvericin, allobeauvericin, enniatin and beauvenniatin analogues discussed and identified in this study.

The higher fungi (*Dikarya*) constitute one of the largest groups of microbial eukaryotes, responsible for producing numerous bioactive secondary metabolites. A diverse set of fungi belonging to the *Hypocreales* order synthesize non-ribosomal cyclic peptides [1,2]. Some of these have been recognized as emerging toxins and agents of competition between divergent taxa. In particular, the entomopathogenic genera *Beauveria*, *Isaria* and *Cordyceps* as well as various phytopathogenic *Fusarium* spp. are known to biosynthesize and accumulate different depsipeptides like beauvericins (BEAs), beauvenniatins (BEAEs) or enniatins (ENNs) [3,4,5,6,7,8,9]. ENNs, BEAs and BEAEs are structurally related mycotoxins consisting most often of three alternating D-2-hydroxyisovaleric (D-Hiv) acid and three *N*-methyl L-amino acid residues (Figure 1) [1,2,10,11]. However, in some BEA and BEAE congeners, one to three D-Hiv units are exchanged for 2-hydroxyisocaproic acid (D-Hmp), e.g., BEA A, B, C, F or BEAE G_1_, G_2_, G_3_. Moreover, yet another hydroxy acid, D-2-hydroxybutyric acid (D-Hbu), may take part in depsipeptides, e.g., in BEA G_1_ and BEA G_2_ [10,11,12,13]. The cyclodepsipeptides most frequently investigated and detected in foods and feeds are enniatin A (ENN A), A_1_ (ENN A_1_), B (ENN B), B_1_ (ENN B_1_) and beauvericin (BEA) (Figure 1) [5,7,14,15,16,17].

BEAs, BEAEs and ENNs exhibit cytotoxic effects to mammalian and human cell lines, inducing apoptosis and DNA fragmentation. They show activity against human cancer cell lines by triggering apoptotic pathways and inhibiting cancer cell motility [10,13,18,19,20,21]. The toxicity of these cyclodepsipeptides is associated with their impact on ion transport through cell membranes [18,22,23]. They exhibit antibacterial and antifungal properties and inhibit the growth of *Mycobacterium tuberculosis* as well as *Plasmodium falciparum* [10,12,24,25,26]. Many cyclodepsipeptides are of pharmaceutical interest, due to their biological activities and possible anticancer effects [18,20,27]. On the other hand, these compounds frequently contaminate cereal products. Thus, their full impact on human and animal health remains largely unexplored [17,28,29,30].

As a rule, non-ribosomal peptide synthesis occurs through a mechanism of multistep condensation catalyzed by non-ribosomal peptide synthases (NRPSs) [31,32]. These are modularly organized multienzyme complexes, in which each module is responsible for the elongation of a growing molecule either proteinogenic or non-protein amino acids, as well as carboxyl and hydroxy acids. Each module of the NRPS system is composed of several domains, which themselves carry out distinct catalytic activities and can be characterized by conserved, core motifs of functional importance (e.g., catalytic sites) [32,33,34].

Some depsipeptide synthases have already been characterized in vitro. The enniatin synthase (ESYN1, 347 kDa), which was described by Zocher et al. from *Fusarium oxysporum*, catalyzed the enniatin synthesis [35]. The production of ENNs is carried out by the condensation of three dipeptidol units through cyclization. ESYN1 synthesizes enniatins from amino acid precursors, mainly, valine, leucine and isoleucine, moreover, from D-2- hydroxyisovaleric acid and S-adenosylmethionine. In the individual functional modules, two adenylation domains are responsible for the specific recruitment of the substrates, respectively, D-2-hydroxyisovaleric acid and L-amino acid, both activated as acyl adenylate intermediates [36,37,38,39,40]. The entire biosynthetic gene cluster responsible for beauvericin production, from entomopathogen—*Beauveria bassiana*—has been characterized [33,41]. Moreover, a few years later, the beauvericin synthase (FpBEAS) from *Fusarium proliferatum* was also described [34]. The elucidated beauvericin synthase (*bbBEAS*) gene encodes a single polypeptide chain with a molecular mass of about 351 kDa [33]. Similarly to ENN biosynthesis, BEA is also assembled by a thiol template mechanism [33,34]. Nevertheless, ESYN1 and BEAS can diverge in selection of substrate needed for chain construction. Beauvericin synthase adenylation domains preferably utilize amino acid residues like *N*-methyl-L-phenylalanine, *N*-methyl-L-leucine, *N*-methyl-L-norleucine or *N*-methyl-L-isoleucine [41].

Significant sequence similarities between the enniatin and beauvericin synthases were found, where BEAS from *B. bassiana* showed 60% identity to ESYN from *F. equiseti* [33]. Indeed, some *Fusarium* species like *F. proliferatum*, *F. poae*, or *F. oxysporum*, isolated from infected field samples, have been described as beauvericin and enniatin producers simultaneously, which can be explained by both cyclodepsipeptides having a common metabolic pathway [7,16,17,29,42].

In the present work, we investigated the cyclodepsipeptide (ENN, BEA and BEAE) production among selected entomopathogenic (*Beauveria*, *Cordyceps*, *Isaria*), as well as phytopathogenic (*Fusarium*) fungi genotypes, representing the ecologically diverse *Hypocreales* order. The results of bioinformatic and phylogenetic analyses allowed us to pinpoint novel strains of cyclodepsipeptide producers and place their chemotypes into the context of extant genetic diversity.

## 2. Results and Discussion

### 2.1. Fungal Species Identification

Nineteen fungal strains were identified and used in the study. Some of the analyzed strains represented a group of plant pathogens (nine *Fusarium* strains belonging to six species), often isolated from such crop species as wheat, rye, pea or asparagus [42]. The second group of identified fungi consisted of selected strains representing the well-known entomopathogenic genera. Among these, representatives of *Beauveria*, *Cordyceps* and *Isaria* genera were present. All the above genera are comprised of species largely characterized as the broad host range facultative entomopathogens, important for control of insect populations in nature and with a possible use in biocontrol [43,44,45]. As such, they are frequently considered as a possible microbial insecticide against insects feeding on crops, including lepidopterans and coleopterans [46,47,48].

The molecular identification of all fungal strains was performed using sequence analysis of the obtained amplicons from polymerase chain reaction (PCR). The DNA regions were amplified with ITS4/ITS5 primers (for entomopathogens) and Ef728M/TefR1 primers (for phytopathogens) and were subsequently sequenced. To confirm species identification, amplified DNA fragments were compared with reference genes from the GenBank Database.

**Figure 2:**
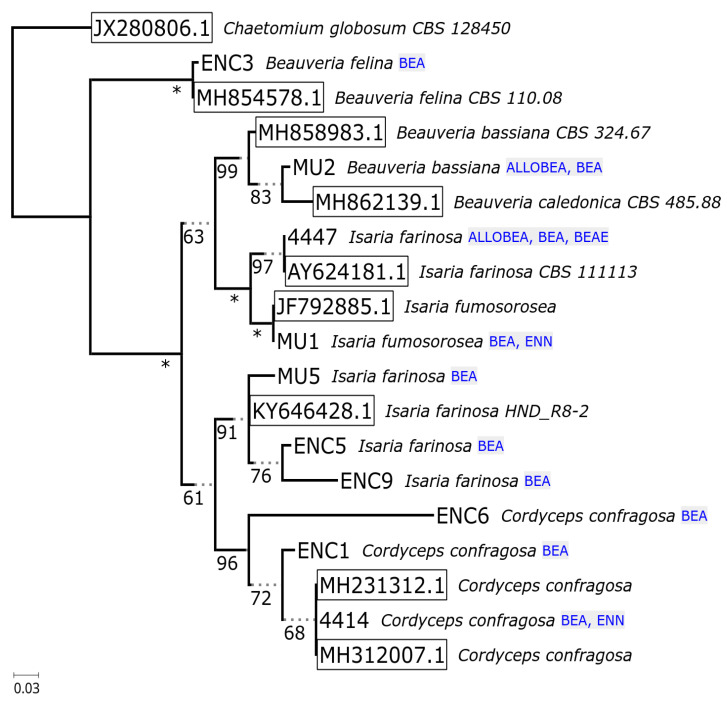
Phylogeny of 19 ITS rDNA partial sequences. Numbers below branches indicate support (‘*’ indicates total support for bipartition), box outlines mark reference sequences, and annotated compound classes are listed beside the leaves. The majority-rule consensus tree was based on the alignment of 475 columns with 117 parsimony informative sites. The TIM3e + G4 model was chosen by IQTREE based on BIC criterion. A total of 103 ultrafast bootstrap iterations were carried out (automated stopping criterion for tree search). All bipartitions with below 50% ultrafast bootstrap support were collapsed in the final representation.

**Figure 3:**
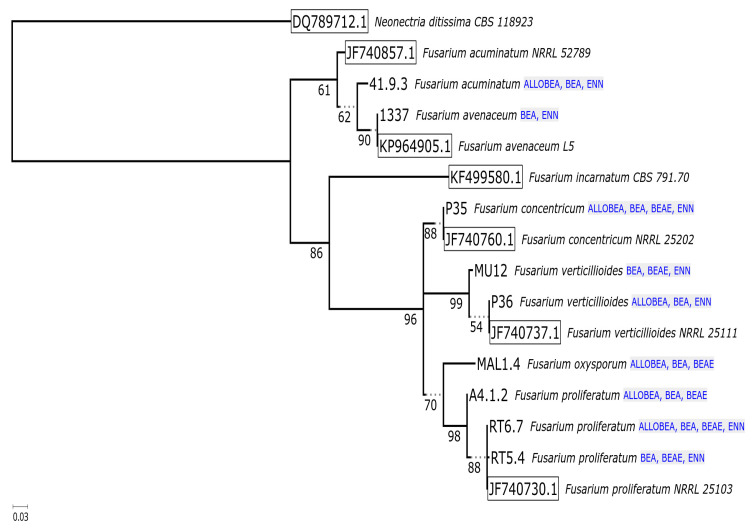
Phylogeny of 16 *tef*-1α partial sequences. Numbers below branches indicate support, box outlines mark reference sequences, and annotated compound classes are listed beside the leaves. The majority-rule consensus tree was based on the alignment of 435 columns (including 87 coding triplets) with 101 parsimony informative sites. The following models were chosen by IQTREE based on BIC criterion: TN + F + G4 for intronic fragments and MG + F3X4 + I for coding parts. A total of 102 ultrafast bootstrap iterations were carried out (automated stopping criterion for tree search). All bipartitions with below 50% ultrafast bootstrap support were collapsed in the final representation.

Acquired sequences by the amplification of ITS and *tef*-1α fragments from the investigated fungal strains showed over 98% similarity to the reference genes. The high identity level to the reference sequences from GenBank showed that all fungal species have been identified correctly. Results of molecular identification and characterization of fungal strains are described in Table 1. Relationships between examined isolates were ascertained as maximum likelihood phylogenetic tree reconstructions based on the ITS1 and *tef*-1α sequences. For phylogeny of the internal transcribed spacer (Figure 2), the additional reference sequences of *Chaetomium globosum* (JX280806.1), *Beauveria felina* (MH854578.1), *Beauveria bassiana* (MH858983.1), *Isaria farinosa* (AY624181.1; KY64628.1), *Isaria fumosorosea* (JF792885.1) and *Cordyceps confragosa* (MH231312.1; MH312007.1) were included in the analysis. For the reconstruction based on *tef*-1α marker (Figure 3), the sequences of *Neonectria ditissima* (DQ789712.1), *Fusarium acuminatum* (JF740857.1), *Fusarium avenaceum* (KP964905.1), *Fusarium incarnatum* (KF499580.1), *Fusarium concentricum* (JF740760.1), *Fusarium verticillioides* (JF740737.1) and *Fusarium proliferatum* (JF740730.1) were included in the analysis.

The analyses of the ITS and *tef*-1α genes have been frequently employed in the phylogenetic studies of fungi belonging to the *Hypocreales* order [3,49,50,51,52,53]. However, based only on these conserved taxonomic markers, the determination of differences between closely related genotypes is often limited. The analysis of secondary metabolite biosynthesis-related genes’ sequences, revealing the intraspecific and interspecific polymorphisms in datasets comprised of isolates from multiple species/genera, is often a better solution [5,42,54].

### 2.2. Non-Ribosomal Peptide Synthetase Genes Divergence

The phylogeny reconstructions of housekeeping sequences (ITS—Figure 2, *tef*-1α—Figure 3) point to the ancestral biosynthesis of beauvericin independently diverging into biosynthesis of other compounds (enniatins, beauvenniatins, allobeauvericins) across different taxa. The evolutionary relationships suggested by beauvericin gene fragments seem to support the notion of *B. felina* as an early diverging beauvericin producer, however, the obtained sequence fragment closely matches the cyclosporin synthase recently elucidated by Xu et al. [55], so verification by cloning, functional genomics and/or whole genome sequencing is likely needed to fully verify the synthase sequence. The divergent positioning of three *Fusarium* isolates (1337, 41.9.3, P36) as well as an unusual GenBank reference sequence for *F. oxysporum f.sp. cucumerinum* (itself highly similar to the known enniatin synthase from *F. scirpi*, accession Z18755) raises the possibility of parallel acquisition of cyclic depsipeptide synthases in ancient complexes within *Fusarium* genus.

**Figure 4:**
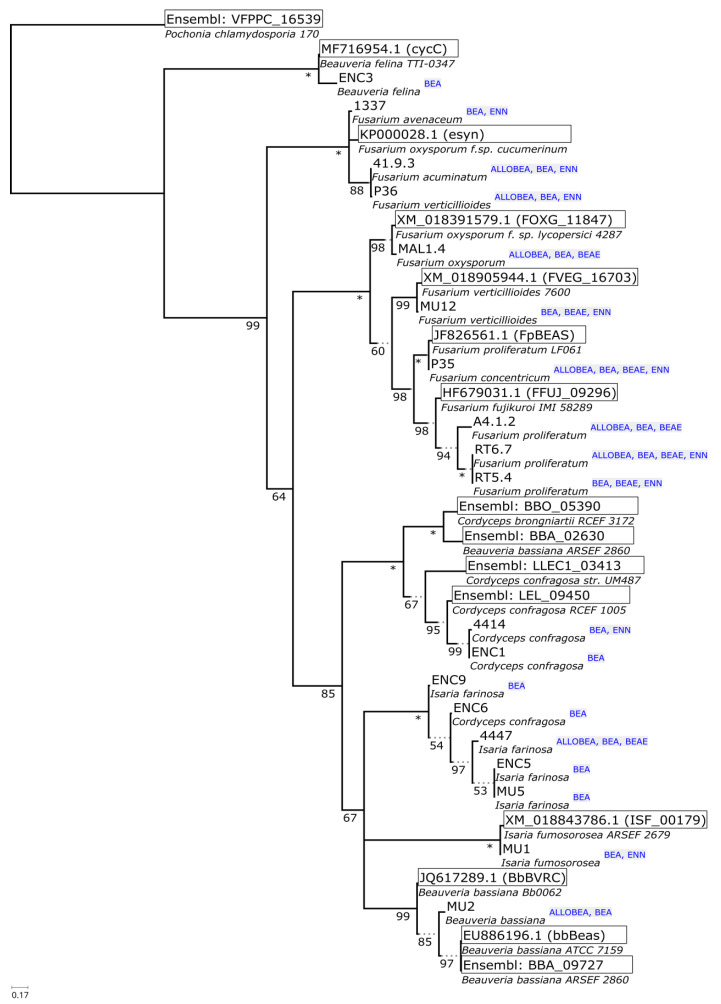
Phylogeny of 34 partial sequences of putative *BEAS* amplicons. Numbers below branches indicate support (‘*’ indicates total support for bipartition), box outlines mark reference sequences from GenBank and Ensembl/Fungi, annotated compound classes are listed beside the leaves. The majority-rule consensus tree was based on the alignment of 157 codons with 147 parsimony informative codons. The MGK + F3X4 + G4 model was automatically fit by IQTREE based on BIC criterion. A total of 115 ultrafast bootstrap iterations were carried out (automated stopping criterion for tree search). All bipartitions with below 50% ultrafast bootstrap support were collapsed in the final representation.

Notably, such separation of two diverging variants of the synthase was previously observed in our earlier parsimony-based reconstruction [42], where predominantly enniatin-producing *F. avenaceum/acuminatum/tricinctum* isolates clustered distinctly separate from beauvericin-producing strains. Regardless of this, save for the *B. felina* sequence, all Cordycipitaceae isolate sequences form a well-supported clade with distinct separation between bassianolide synthase-like sequences (canonical bassianolide synthase from *B. bassiana*, model sequences from *C. confragosa* and *C. brongniartii*, isolates 4414 and ENC4) and beauvericin synthase-like fragments (canonical beauvericin synthase from *B. bassiana*, sequences from isolates tentatively identified as *I. farinosa*, *I. fumosorosea*, as well as single *C. confragosa* isolate ENC6). It is worth noting that the fairly recent update of Cordycipitaceae nomenclature and phylogeny shows that past identification of even reference *Isaria/Cordyceps* strains available from well-curated collections was not well supported by newly available molecular data [56]. In the context of continued research in the field [57], *I. farinosa* in particular is demonstrated to be a wide label spanning different species within the redefined, monophyletic *Cordyceps* and *Akanthomyces* genera (Figure 4).

With regards to the sequencing and phylogeny of putative *BEAS* homolog fragments, the validation of these sequences on the level of full genomic sequence and functional experiments is a desired follow-up. In particular, the further study of the ENC3 *Beauveria felina* isolate on genomic and functional levels is likely to provide insight as to the diversification of beauvericin/beauvenniatin/enniatin biosynthesis across this important genus of insect pathogens (Figure 4).

### 2.3. In Vitro Cyclodepsipeptide Biosynthesis

Quantitative analysis of ENN A (compound **12** in Table 1), ENN A_1_ (**13**), ENN B (**14**), ENN B_1_ (**15**) and BEA (**1**) was conducted using LC-MS/MS (Table 2). Beauvericin was produced by all investigated fungi, with the most efficient producers being *Fusarium* strains, such as *F. proliferatum* (RT6.7, RT 5.4) and *F. concentricum* (P35). To some extent, all of the studied fungi are known producers of beauvericin [42], however, it is important to note that each fungal strain possesses unique abilities to produce different amounts of BEA, which can also be affected by the cultivation conditions [58,59].

In contrast, the most efficient producers of the four enniatin analogues were *F. acuminatum* (41/9/3) and *F. avenaceum* (1337). The production of enniatins by phytopathogenic fungi belonging to the genus *Fusarium* has been described earlier [5,6,7,17,28,29,30,42]. The most efficient enniatin producer among entomopathogenic fungi was the isolate of *C. confragosa* (4414). Notably, some of the investigated fungal strains were not able to biosynthesize any of the four enniatins, and this group included entomopathogenic fungi *B. bassiana* (MU3), *B. felina* (ENC3), *I. farinosa* (4447, ENC5, ENC9, MU5) and *C. confragosa* (ENC1, ENC6). The same entomopathogenic fungal species were previously only investigated for beauvericin production, or production of beauvericin analogues [9,11,12,13]. Indeed, we were not able to find any previous reports concerning the production of enniatins by those entomopathogenic fungi. On the contrary, our own analyses showed that for at least two strains—*I. fumosorosea* (MU1) and *C. confragosa* (4414)—a limited capacity to produce enniatins exists, as each isolate was able to synthesize two of the four enniatin analogues (Table 2). Moreover, the two phytopathogenic fungal species *F. proliferatum* and *F. oxysporum* did not synthesize enniatin analogues, although they have earlier been described as enniatin producers [16,29,42,60]. This suggests a need for comprehensive reevaluation of cyclodepsipeptide profiles across multiple representatives of known producing taxa, in particular as the production of enniatins may depend on cultivation conditions, storage conditions or the host and the environment from which the fungi were isolated [59].

Liquid chromatography–ion-trap mass spectrometry (LC-ITMS) was used for the analysis of BEAs and BEAEs appearance in all investigated fungal crude extracts (Table 3). Two selected strains (P35 and 4447) were also analyzed using liquid chromatography–high-resolution mass spectrometry (LC-HRMS) for accurate mass measurements and HRMS/MS (Table 4). We have previously tentatively shown that these two strains produce BEA A, BEA C, BEA D, BEA F, BEA J, BEA K, BEAE A, and BEAE L [3]. Only five of the studied strains (P35, RT 6.7, 4447, MAL 1.4, and A4.12) were found to produce a wide range of different BEA and BEAE analogues (>15) (Table 3, Figure 1).

**Figure 5:**
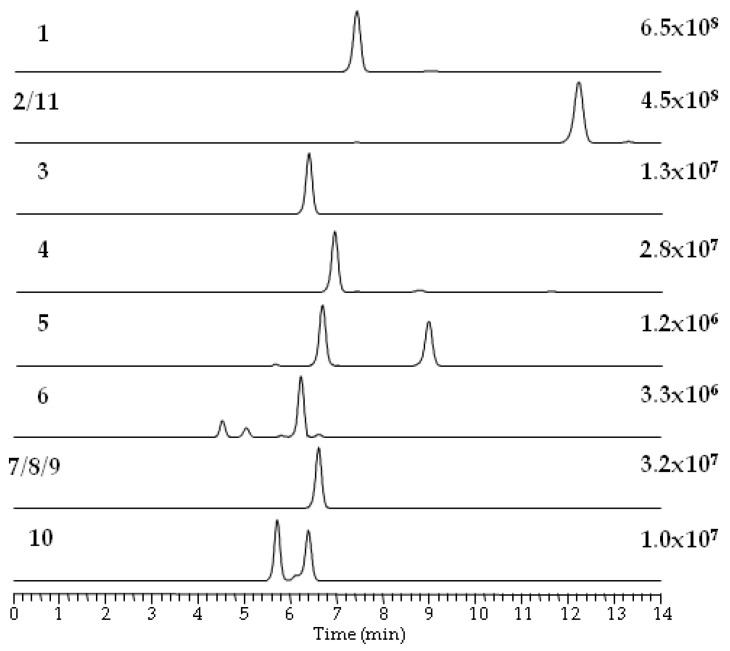
Extracted ion LC-HRMS chromatograms (±5 ppm) of the [M + NH_4_]^+^ ions for beauvericin, allobeauvericin and beauvenniatin analogues in the crude extract from rice cultures of *Fusarium concentricum* (P35) and *Isaria farinosa* (4447). The numbers refer to the compounds in Figure 1.

Using reversed-phase liquid chromatography (RPLC), the elution of individual BEAs and BEAEs was highly related to their molecular weights, i.e., beauvericin analogues with a higher molecular weight eluted later (Table 4). The presumed BEAs and BEAEs provided plentiful [M + NH_4_]^+^ ions during electrospray ionization, while [M + H]^+^ and [M + Na]^+^ ions accounted for only few percent of the total ion yield. In total, five beauvericins (compounds **1**–**5**), five beauvenniatins (**6**–**10**) and one allobeauvericin (**11**) were tentatively detected in different fungal extracts (Figure 1 and Figure 5, and Table 4).

**Figure 6:**
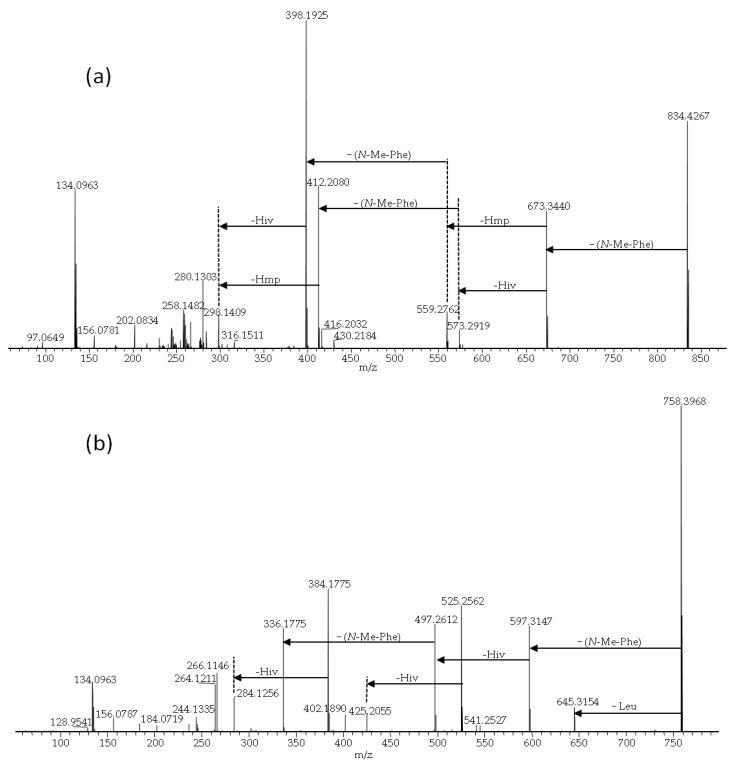
LC-HRMS/MS spectra from higher collision dissociation of the [M + Na]^+^ ions of putative beauvericin analogues: beauvericin B (**a**) and beauvericin E (**b**).

**Figure 7:**
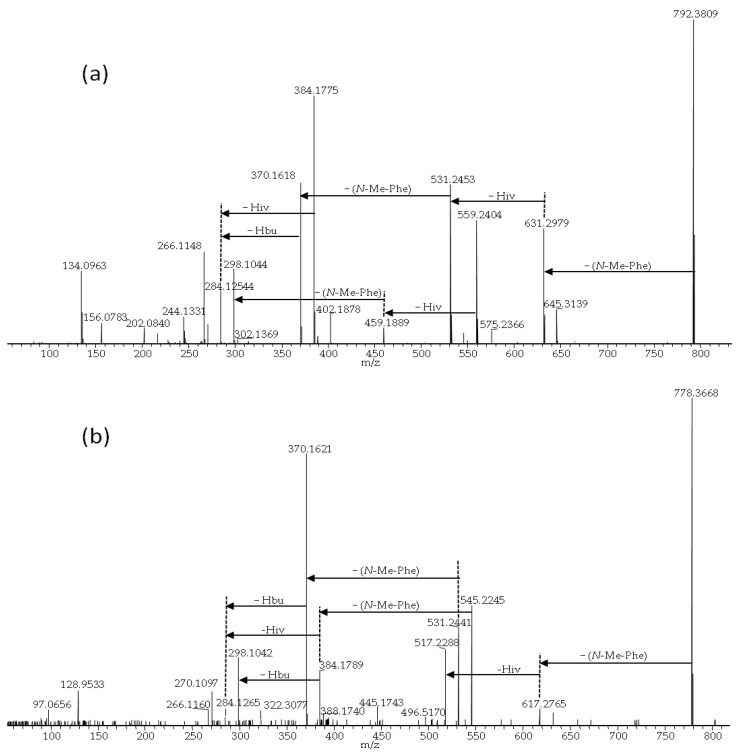
LC-HRMS/MS spectra from higher collision dissociation of the [M + Na]^+^ ions of putative beauvericin analogues: beauvericin G_1_ (**a**) and beauvericin G_2_ (**b**).

BEA itself was the most common analogue and was produced in the highest relative amounts (in terms of LC-MS peak areas) by all studied fungal strains. We used BEA (**1**) as a reference for comparing product-ion spectra of the sodiated ions of different analogues [3]. All BEA analogues, characterized by the presence of *N*-methyl-phenylalanine in their structure, were tentatively identified by the presence of a product-ion due to a loss of −161 Da. The beauvericins that were observed in the studied strains varied in hydroxy acid composition (Figure 6a,b). BEA E (**3**) contains one leucine moiety in addition to two *N*-methyl-phenylalanine units, and this analogue was tentatively identified by the presence of a product-ion corresponding to a loss of −127 Da. BEA E (**3**) possesses three D-Hiv units giving rise to product-ions corresponding to a loss of −100 Da (Figure 6b) [11,24]. In contrast, BEA B (**2**) possesses two D-Hmp units and one D-Hiv unit, giving rise to product-ions corresponding to −114 and −100 Da, respectively (Figure 6a) [12]. Furthermore, BEA G_1_, and G_2_ (**4**, **5**) consist of one or two D-Hbu groups, respectively, which can be observed by product-ions, corresponding to a loss of −86 Da (Figure 7a,b) [13].

**Figure 8:**
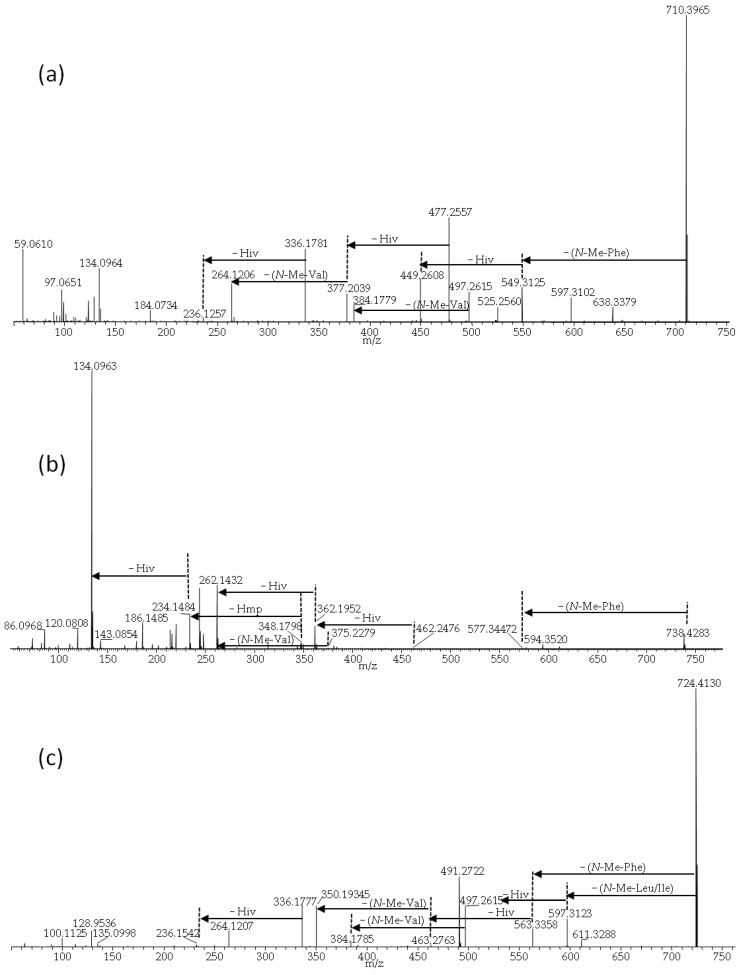
LC-HRMS/MS spectra from higher collision dissociation of the [M + Na]^+^ ions of beauvenniatin analogues: beauvenniatin B (**a**), beauvenniatin G_1_/G_2_/ G_3_ (**b**) and beauvenniatin * (**c**).

ALLOBEAs A, B (**11**) and C are isomers of BEAs A/F, B (**2**) and C, which are composed of three *N*-methyl-phenylalanine moieties and one, two or three D-Hmp groups, respectively [12]. BEAEs **7**–**9**, which are also isomers [10], were produced in the smallest relative amounts. The HRMS/MS spectra of their sodiated ions were of poor signal/noise ratios; still, they were better than spectra of their ammoniated ions. The detected isomers of depsipeptides consist of the same moieties, therefore, we are not able to tell which isomer/s is/are present in the sample (Figure 5). The differences between studied compounds (**2**/**11** and **7**/**8**/**9**) were described earlier, on the basis of NMR analyses [10,12]. All investigated BEAEs (**6**–**10**) contain one group of *N*-methyl-valine and two *N*-methyl-phenylalanine residues, which was manifested by product-ions corresponding to losses of −161 and −113 Da (Figure 8a–c). BEAE B (**6**) contains three D-Hiv groups (Figure 8a) [10], while BEAEs **7**–**9** (Figure 8b) appeared to contain one D-Hmp group instead of D-Hiv, based on our HRMS/MS data. Moreover, we detected one compound (**10**) in the crude extract from *F. concentricum* (P35) pure rice cultures that appeared to be composed of an unreported combination of amino and hydroxy acids. Our HRMS/MS data showed that it could be an unreported BEAE consisting of three D-Hiv groups, one *N*-methyl-valine, *N*-methyl-phenylalanine and *N*-methyl-leucine or -isoleucine group (Figure 8c). The latter two cannot be distinguished by mass spectrometry alone. For future investigations, nuclear magnetic resonance (NMR) will be considered in order to better discern the actual depsipeptide structure.

## 3. Materials and Methods

### 3.1. Fungal Strains, Media and Growth Conditions

Fungal strains from the *Hypocreales* order were isolated from infected plants and insects found in Poland. All isolated strains were placed in the fungi collection of the Institute of Plant Genetics, Polish Academy of Sciences, Poznań, Poland. For characterization of fungal isolates, the species, strain number and host species have been summarized in Table 1. For genomic DNA extraction, the growing mycelia of individual *Hypocreales* fungi were purified and cultivated on potato dextrose agar medium (PDA, Oxoid, Basingstoke, UK) for seven days at controlled conditions (20/25 °C, 12 h photoperiod). Harvested mycelia were stored at −20 °C. For qualitative and quantitative cyclodepsipeptide analyses, fourteen day old pure rice cultures of each fungal species were used, according to the procedure described previously [53].

### 3.2. DNA Extraction, Molecular Identification, PCR Primers and DNA Sequencing

Genomic DNA extraction from fungal samples was performed using a modified method with the CTAB (hexadecyltrimethylammonium bromide), according to Gorczyca et al. [61]. The DNA concentration in extracts was quantified using an ND-1000 spectrophotometer (Thermo Scientific, West Palm Beach, FL, USA) and stored in a freezer at −20 °C. For *Fusarium* species identification, the sequence analysis of a variable fragment of the translation elongation factor 1α gene (tef-1α) was performed, whereas for the other species, the molecular identification was carried out on the basis of the sequence analysis of the internal transcribed spacers of the ribosomal DNA region (ITS1–ITS2).

Polymerase chain reactions (PCRs) were carried out using DreamTaq Green DNA polymerase (Thermo Scientific, Espoo, Finland). The conditions for PCR amplification of the tef-1α gene fragment were described earlier by Gálvez et al. [28], where primers Ef728M (5′-CATCGAGAAGTTCGAGAAGG-3′) and TefR1 (5′-GCCATCCTTGGAGATACCAGC-3′) [62] were used. The conditions for the PCR amplification of the ITS1–ITS2 DNA region were described earlier by Kozłowska et al. [51], where primers ITS4 (5′-TCCTCCGCTTATTGATATGC-3′) and ITS5 (5′-GGAAGTAAAAGTCGTAACAAGG-3′) [63] were used. Obtained sequences from PCR reaction were separated in 1.5% agarose gel (EURx, Gdańsk, Poland) with GelGreen Nucleic Acid Stain (Biotium, Inc., San Francisco, CA, USA).

The beauvericin synthase gene (*BEAS*) was partially amplified using the beas_1 (5′-TKGARCAGCGBCAYGAGACM-3′) and beas_2 (5′-GGWCGRGGGAARTCRGTDGG-3′) primers designed earlier by Stępień and Waśkiewicz [42].

The PCRs were done in 25 μL volumes containing Color PfuPlus! DNA Polymerase (EURx, Gdańsk, Poland); 2.5 μL of 10 x Pfu Buffer contains 15 mM MgSO4, 0.5 µmol of forward/reverse primers and 0.25 mM of each dNTP and 10–20 ng of fungal DNA. The PCR conditions were as follows: 5 min at 95 °C, 35 cycles of 30 s at 95 °C, 30 s at 61 °C, 1 min at 72 °C and 7 min at 72 °C.

In order to analyze the sequences, amplicons were purified with exonuclease I (Thermo Scientific, Espoo, Finland) and FastAP shrimp alkaline phosphatase (Thermo Scientific, Espoo, Finland), afterwards they were labeled using forward primer and the BigDyeTerminator 3.1 kit (Applied Biosystems, Foster City, CA, USA). Finally, PCR-amplified DNA fragments were precipitated with 96% ethanol according to Tomczyk et al. [49].

### 3.3. Sequence Analysis and Phylogeny Reconstruction

Individual DNA sequences were aligned using MUSCLE 3.8.31, available via SeaView 4.6.4 environment [64] (Linux 64bit version). In the case of the *BEAS* coding sequence, the sequences were first translated and aligned as proteins, then backtranslated into codon alignments. In the case of TEF, exon boundaries were corrected manually to match known splicing site consensus (GT-AG type introns). To investigate grouping of obtained sequences, their top BLAST hits (discontiguous MegaBLAST was performed on the non-redundant GenBank database through NCBI/BLAST stand-alone service, accessed on 22 May 2019) were trimmed appropriately and were also included in the final alignments. In the case of *BEAS* homologs, additional *Beauveria*/*Cordyceps* sensu lato representatives were retrieved from Ensembl/Fungi database (accessed on 17 May 2019).

The phylogeny reconstructions were carried out with IQTREE 1.6.9 [65] (Linux 64-bit version) using automated model fitting, partitioning and ultrafast bootstrap [66]. During the reconstruction, ITS rDNA was modelled as non-coding DNA sequence, while *BEAS* was investigated using codon-based models. In the case of the translation elongation factor (*tef*-1α), the coding fragment was investigated as a single coding partition, while the DNA model was ascertained for intronic parts present in the amplified fragments.

### 3.4. Mycotoxin Analyses

#### 3.4.1. Chemicals

Mycotoxin standards (>99%) (enniatins A, A_1_, B, B_1_ and beauvericin), ammonium formate (>99.99%) and methanol (HPLC grade > 99.9%) were purchased from Sigma-Aldrich (St. Louis, MO, USA). Standard solutions of ENNs (2.0 ng/μL of each analogue) and BEA (1.0 ng/μL) were prepared in methanol and kept in a freezer at −20 °C. Acetonitrile and water for LC-MS were from Thermo Fisher Scientific (Optima LC-MS grade, Waltham, MA, USA).

#### 3.4.2. Extraction and purification

For screening the fungal rice cultures for the presence of different depsipeptide analogues using LC-ion-trap MS, 0.15 g aliquots weighed (after lyophilized and ground into powder) of each culture were extracted using 1.5 mL of methanol/water (9:1, *v*/*v*) by shaking on an orbital shaker (225 min^−1^, 90 min) and by sonication for 20 min. After centrifugation using a Beckman J2-MC centrifuge (Beckman Coulter Inc., Fullerton, CA) at 15,000× *g*, for 10 min, extracts were filtered through a 0.22 µm nylon membrane (Costar, Corning Inc., Corning, NY, USA) and transferred to chromatography vials. For quantification of mycotoxins (BEA, ENNs) by LC-MS/MS, individual culture aliquots (1 g) were mixed with 2 mL of acetonitrile-water (9:1, *v*/*v*) and after homogenization (homogenizer H500, Pol-Ekoaparatura, Poland), centrifugation (at 4500× *g* for 5 min), and filtration (0.20 µm syringe filter—Chromafil, Macherey-Nagel, Duren, Germany) were prepared for chromatographic analysis.

#### 3.4.3. Liquid Chromatography Mass Spectrometry Analyses

Culture extracts were screened for the presence of ENNs, BEA, BEAE and ALLOBEA analogues using an LCQ Fleet ion trap mass spectrometer (Thermo Fisher Scientific) coupled to a Waters Acquity UPLC (Milford, MA, USA) with chromatographic column—SunFire C18; instrumental conditions were described earlier by Urbaniak et al. 2019 [3].

Quantitative analysis of mycotoxins (BEA and ENNs) were performed using an LC-MS/MS instrument consisting of an UPLC™ system (Acquity, Waters, Milford, MA, USA) coupled to a triple quadrupole mass spectrometer (TQD, Waters Micromass, Manchester, UK), equipped with an electrospray ionization interface according to Stanciu et al. [67] with our own modifications, described below. The separation of beauvericin and enniatins was achieved using a BEH C18 chromatographic column (100 × 2.1 mm i.d., 1.7 µm particle size; Waters) with injection volume—3 µL and flow rate of mobile phase—0.3 mL/min. The mobile phase consisted of water (A) and methanol (B), both containing 5 mM ammonium formate and 0.1% (*v*/*v*) formic acid. The gradient program was as follows: initial conditions at 80% A, 20% B for 2 min; then, from 20% to 90% B in 5 min; next, 90% B for 6 min; from 90% to 100% B in 3 min; return to initial conditions in 2 min. For data processing, Empower^TM^ 1 software was used (Waters, Manchester, UK). Chromatographic parameters are shown in Table 5.

#### 3.4.4. Liquid Chromatography High-Resolution Mass Spectrometry (HRMS)

A Q-Exactive Fourier-transform high-resolution mass spectrometer (Thermo Fisher Scientific) coupled to a Vanquish Horizon UHPLC (Thermo Fisher Scientific) was used to acquire accurate mass data and high-resolution product-ion spectra of putative beauvericin analogues. Separation was achieved on a Kinetex C18 column (75 × 2.1 mm; 2.6 µm particles; Phenomenex, Torrance, CA, USA) held at 30 °C with the injection volume—3 µL and flow rate—0.3 mL/min. The mobile phase consisted of line A (5 mM ammonium formate) and line B (5 mM of ammonium formate in MeOH/water, 95:5, *v*/*v*) with gradient program as follows: 0–15 min, 75% B; 15–15.1 min, 80% B; 15.1–18 min, 100% B; 18.1–21 min, 75%. The instrument was run in the full-scan mode (*m*/*z* 600–900). The fragmentation of the sodiated molecular ions (MS/MS) from eleven metabolites was performed in the full-scan mode (m/z 150–1200), according to the previous work [3]. Elemental compositions were calculated using Xcalibur, version 2.3. (Thermo Fisher Scientific).

## 4. Conclusions

The present study demonstrates the high variability of naturally produced cyclohexadepsipeptides by *Hypocreales* fungi. It also emphasizes that different fungi have independently evolved a significant level of divergence inside the non-ribosomal peptide synthase genes involved in depsipeptide biosynthesis, along with functional adaptation towards biosynthesis of overlapping yet diversified metabolite profiles. Additionally, a new compound has been described as a naturally occurring beauvenniatin analogue (**10**).

## Figures and Tables

**Table 1:** *Hypocreales* fungi used in this study, host species, fungal strain identification based on ITS1–ITS2 or *tef*-1α sequence analysis and comparison with reference sequences from the GenBank Database.

No.	Strain	Species	Host	Sequence Nucleotide Identity
1	1337	*Fusarium avenaceum*	wheat	99.76% identity to the *Fusarium avenaceum* acc. number KP964905.1
			(*Triticum* L.)	
2	MU2	*Beauveria bassiana*	bark beetle	100% identity to the *Beauveria bassiana* acc. number KU158425.1
			(*Trypodendron lineatum*)	
3	ENC3	* Beauveria felina *	caterpillar	98.37% identity to the *Beauveria felina* acc. number MH854578.1
			(*Lepidoptera* sp.)	
4	15222	*Fusarium acuminatum*	pea	98.19% identity to the *Fusarium acuminatum* acc. number JF740857.1
			(*Pisum* L.)	
5	P35	*Fusarium concentricum*	pineapple	100% identity to the *Fusarium concentricum*, acc. number JF740760.1
			(*Ananas comosus*)	
6	RT6.7	*Fusarium proliferatum*	rice	99.78% identity to the *Fusarium proliferatum* acc. number JF740730.1
			(*Oryza sativa*)	
7	RT5.4	*Fusarium proliferatum*	rice	99.78% identity to the *Fusarium proliferatum* acc. number JF740730.1
			(*Oryza sativa*)	
8	MU12	*Fusarium verticillioides*	banana	98.66% identity to the *Fusarium verticillioides* acc. number JF740717.1
			(*Musa* L.)	
9	P36	*Fusarium verticillioides*	pineapple	100% identity to the *Fusarium verticillioides* acc. number JF740717.1
			(*Ananas comosus*)	
10	4447	* Isaria farinosa *	bark beetle	100% identity to the *Isaria farinosa*, acc. number AY624181.1
			(*Trypodendron lineatum*)	
11	ENC5	* Isaria farinosa *	bark beetle	98.92% identity to the *Isaria farinosa*, acc. number KY646428.1
			(*Trypodendron lineatum*)	
12	ENC9	* Isaria farinosa *	beetle	98.89% identity to the *Isaria farinosa*, acc. number DQ888729.1
			(*Coleoptera* sp.)	
13	MU5	* Isaria farinosa *	pine beetle	99.53% identity to the *Isaria farinosa*, acc. number KY646428.1
			(*Dendroctonus ponderosae*)	
14	MU1	* Isaria fumosorosea *	beetle	98.38% identity to the *Isaria fumosorosea*, acc. number JF792885.1
			(*Coleoptera* sp.)	
15	4414	* Cordyceps confragosa *	parent bug	100% identity to the *Cordyceps confragosa*, acc. number MH231312.1
			(*Elasmucha* sp.)	
16	ENC1	* Cordyceps confragosa *	bark beetle	98.08% identity to the *Cordyceps confragosa*, acc. number MH312006.1
			(*Trypodendron lineatum*)	
17	ENC6	* Cordyceps confragosa *	caterpillar	99.82% identity to the *Cordyceps confragosa*, acc. number MH312006.1
			(*Lepidoptera* sp.)	
18	MAL 1.4	*Fusarium oxysporum*	asparagus	100% identity to the *Fusarium oxysporum* acc. number KP964890.1
			(*Asparagus officinalis* L.)	
19	A 4.12	*Fusarium proliferatum*	wheat	100% identity to the *Fusarium proliferatum* acc. number KU939029.1
			(*Triticum* L.)	

**Table 2:** Mean concentrations (±standard deviations) of beauvericin and enniatins A, A_1_, B, and B_1_ [ng/g] produced in vitro by 19 fungal strains belonging to 11 species.

Strain	BEA [ng/g]	ENN A [ng/g]	ENN A_1_ [ng/g]	ENN B [ng/g]	ENN B_1_ [ng/g]
1337 (Fav)	49.3 ± 5.6	4300 ± 160	63800 ± 2430	ND	ND
MU2 (Bb)	389 ± 22.4	ND	ND	ND	ND
ENC3 (Bf)	211 ± 12	ND	ND	ND	ND
41/9/3 (Fac)	150 ± 10	56600 ± 1960	68970 ± 2535	ND	ND
P35 (Fco)	130400 ± 2300	127 ± 20	ND	350 ± 37	ND
RT6.7 (Fpr)	135540 ± 3225	ND	ND	1983 ± 37.4	ND
RT5.4 (Fpr)	55420 ± 411	ND	ND	2130 ± 130	ND
MU12 (Fve)	125 ± 4.3	ND	ND	191 ± 12.7	51 ± 4.5
P36 (Fve)	150 ± 8.3	ND	ND	23 ± 2.6	ND
4447 (Ifa)	10240 ± 246	ND	ND	ND	ND
ENC5 (Ifa)	215 ± 15	ND	ND	ND	ND
ENC9 (Ifa)	352 ± 9.6	ND	ND	ND	ND
MU5 (Ifa)	155 ± 9.3	ND	ND	ND	ND
MU1 (Ifu)	900 ± 26.2	ND	ND	720 ± 23.4	71 ± 5
4414 (Cco)	125 ± 15.7	5730 ± 157	ND	ND	9130 ± 128
ENC1 (Cco)	128±12	ND	ND	ND	ND
ENC6 (Cco)	115 ± 13	ND	ND	ND	ND
MAL 1.4 (Fox)	433 ± 11.3	ND	ND	ND	ND
A 4.12 (Fpr)	730 ± 60	ND	ND	ND	ND

ND—not detected. Fav—Fusariumm avenaceum, Bb—Beauveria bassiana, Bf—Beauveria felina, Fac—Fusarium acuminatum, Fco—Fusarium concentricum, Fpr—Fusarium proliferatum, Fve—Fusarium verticillioides, Ifa—Isaria farinosa, Ifu—Isaria fumosorosea, Cco—Cordyceps confragosa, Fox—Fusarium oxysporum.

**Table 3:** Qualitative analysis of BEAs, BEAEs and ENNs produced by *Hypocreales* fungi in this study.

No.	Strain	Species	Metabolic Profile
1	1337	*Fusarium avenaceum*	BEA, ENN A, ENN A_1_
2	MU2	* Beauveria bassiana *	BEA, BEA B, BEA C, BEA F/A, ALLOBEA A, ALLOBEA B, ALLOBEA C
3	ENC3	* Beauveria felina *	BEA,
4	15222	*Fusarium acuminatum*	BEA, BEA C, BEA D, BEA G_1_, ALLOBEA C, ENN A, ENN A_1_
5	P35	*Fusarium concentricum*	BEA, BEA B, BEA C, BEA D, BEA E, BEA F/A, BEA J, BEA G_1_, BEA G_2_, BEAE A, BEAE B, BEAE G_1_/G_2_/G_3_, BEAE *, ALLOBEA A, ALLOBEA B, ALLOBEA C, BEAE L, BEA K, BEA L, ENN A, ENN B
6	RT6.7	*Fusarium proliferatum*	BEA, BEA B, BEA C, BEA D, BEA E, BEA F/A, BEA J, BEA G_1_, BEA G_2_, BEAE A, BEAE B, ALLOBEA A, ALLOBEA B, ALLOBEA C, BEAE L, BEA K, ENN B
7	RT5.4	*Fusarium proliferatum*	BEA, BEA D, BEA J, BEA G_1_, BEAE L, ENN B
8	MU12	*Fusarium verticillioides*	BEA, BEA D, BEA G_1_, BEAE A, BEA K, ENN B, ENN B_1_
9	P36	*Fusarium verticillioides*	BEA, BEA C, ALLOBEA C, ENN B
10	4447	* Isaria farinosa *	BEA, BEA B, BEA C, BEA D, BEA E, BEA F/A, BEA J, BEA G_1_, BEA G_2_, BEAE A, ALLOBEA A, ALLOBEA B, ALLOBEA C, BEAE L, BEA K, BEA L
11	ENC5	* Isaria farinosa *	BEA
12	ENC9	* Isaria farinosa *	BEA
13	MU5	* Isaria farinosa *	BEA
14	MU1	* Isaria fumosorosea *	BEA, ENN B, ENN B_1_
15	4414	* Cordyceps confragosa *	BEA, ENN A, ENN B_1_
16	ENC1	* Cordyceps confragosa *	BEA
17	ENC6	* Cordyceps confragosa *	BEA
18	MAL 1.4	*Fusarium oxysporum*	BEA, BEA B, BEA C, BEA D, BEA E, BEA F/A, BEA J, BEA G_1_, BEA G_2_, BEAE A, BEAE B, ALLOBEA A, ALLOBEA B, ALLOBEA C, BEAE L
19	A 4.12	*Fusarium proliferatum*	BEA, BEA B, BEA C, BEA D, BEA E, BEA F/A, BEA J, BEA G_1_, BEA G_2_, BEAE A, BEAE B, ALLOBEA A, ALLOBEA B, ALLOBEA C, BEAE L

BEA—beauvericin; BEAE—beauvenniatin; ENN—enniatin; ALLOBEA—allobeauvericin.

**Table 4:** Accurate mass and elemental composition of major ions observed for different beauvericin, allobeauvericin and beauvenniatin analogues from LC-HRMS.

Compound	Measured (m/z) [M + H]^+^	Measured (m/z) [M + NH_4_]^+^	Measured (m/z) [M + Na]^+^	Retention Time (min)	Elemental Composition	Mass Error (ppm) [M + H]^+^	Mass Error (ppm) [M + NH_4_]^+^	Mass Error (ppm) [M + Na]^+^
1	784.4144	801.4426	806.3947	7.4	C_45_H_57_N_3_0_9_	−3.7	−1.6	−5
2/11	812.4446	829.4738	834.4267	12.4	C_47_H_61_N_3_O_9_	−4.9	−1.7	−4.8
3	736.4155	753.4431	758.3968	6.4	C_41_H_57_N_3_O_9_	−2.5	−0.9	−3.3
4	770.3997	787.4274	792.3809	7	C_44_H_55_N_3_O_9_	−2.6	−1	−3.5
5	756.384	773.4124	778.3668	6.7	C_43_H_53_N_3_O_9_	−1.9	−0.2	−1.5
6	688.4184	705.4425	710.3965	6.3	C_37_H_57_N_3_O_9_	1.6	−1.1	−3.9
7/8/9	716.4469	733.4745	738.4263	6.7	C_39_H_61_N_3_O_9_	−2.4	−0.9	−5.8
10	702.4344	719.4581	724.413	5.7	C_38_H_59_N_3_O_9_	2.1	−2	−2.6

**Table 5:** Parent and daughter ions, collision energy and limit of detection (LOD) and quantification (LOQ) (ng/g) for mycotoxins.

Compound	Parent ion (m/z) [M+NH_4_]^+^	Primary Daughter Ion (m/z)	Secondary Daughter Ion (m/z)	Collision Energy (eV)	LOD ^a^ (ng/g)	LOQ ^b^ (ng/g)
ENN A	699.4	228.2 *	210.1	36	2	6
ENN A_1_	685.4	214.2 *	210.1	38	3	9
ENN B	657.3	214.1 *	196.0	40	2	6
ENN B_1_	671.2	228.0 *	214.1	57	2	6
BEA	801.2	784.0	244.1 *	28	1	3

* Transitions used for quantification. ^a^ Limit of detection (LOD). ^b^ Limit of quantification (LOQ).
